# Severe acute respiratory syndrome coronavirus 2 (SARS-CoV-2) antibody prevalence in a healthcare worker population exceeded self-reported infection rates, 2020

**DOI:** 10.1017/ash.2022.9

**Published:** 2022-02-07

**Authors:** Susan E. Hoover, Valerie Reed

**Affiliations:** Sanford Health, Sioux Falls, South Dakota

## Abstract

A study of severe acute respiratory coronavirus virus 2 (SARS-CoV-2) seroprevalence at a healthcare institution prior to the availability of vaccine showed that seroprevalence in the cohort increased over 6 months from 25% to 55%. The number of employees with antibodies was higher than the number who reported an exposure or diagnosis at each time point.

Protecting both healthcare workers and patients in hospitals, clinics, and other healthcare institutions from acquiring or transmitting severe acute respiratory coronavirus virus 2 (SARS-CoV-2) has been a major focus during the coronavirus disease 2019 (COVID-19) pandemic. When testing for antibodies against SARS-CoV-2 became available in early 2020, it was of considerable interest as a possible marker for past infection and current vulnerability, particularly prior to the availability of SARS-CoV-2 vaccines.

We conducted a study of SARS-CoV-2 seroprevalence at our large, nonprofit, healthcare institution starting in June 2020 and ending in January 2021. Our objectives were to use a simple design with a remote consenting process to rapidly learn the prevalence, duration, and rate of acquisition of antibodies to SARS-CoV-2 in this population and to assess the relationship of antibodies to reported infections.

## Methods

### Study design, participants, and setting

The study protocol was reviewed and approved by the Sanford Health Institutional Review Board. Employees of Sanford Health were invited to participate through a company-wide e-mail message, postings on the company intranet, and stories on the internal news service. Enrollment occurred via an electronic consent process. Enrollment was initially confined to those working in areas expected to see many COVID-19 patients and was later expanded to any employee with patient contact. Laboratory workers were also eligible.

Enrollment began in June 2020, a time of relatively low COVID-19 prevalence in our communities. During the study period, the region experienced a surge of cases beginning in August 2020 and peaking in late November. Widespread community transmission occurred during that time, and a large number of COVID-19 patients were admitted to our hospitals. Supplies of personal protective equipment were adequate with conservation measures in place, and adherence to infection prevention protocols was generally good.

### Data collection

Electronic consent and questionnaires were collected through REDCap (Research Electronic Data Capture), a secure electronic data capture tool.^1,2^ Every 60 days, participants were invited to complete a survey asking about current symptoms, date of any interval diagnosis of SARS-CoV-2 infection or COVID-19, date of any interval potential exposure to COVID-19, and where they had been working during the interval (eg, COVID-19 unit, emergency department, obstetrics, laboratory, long-term care facility, or other). They also provided a blood sample for the measurement of antibodies to SARS-CoV-2. These blood samples were collected at Sanford clinical laboratory locations, and the tests were performed according to the laboratory routine using Clinical Laboratory Improvement Amendments (CLIA)–certified assays. The initial assay used by our laboratories was for anti–SARS-CoV-2 spike-protein total antibodies (Siemens Atellica, Siemens, Munich, Germany). In November 2020, the laboratory began using an assay for anti-SARS-CoV-2 spike protein IgG (DiaSorin, Sallugia, Italy).

### Data analysis

We summarized the relationship between reported diagnosis and presence of antibodies over time along with the participant’s work location.

## Results

### Study duration

Vaccines against SARS-CoV-2 became available to our study participants in December 2020. At that point, a question was added to the study questionnaire about the vaccine, and a majority of participants who answered indicated that they would be vaccinated. Because our laboratory used an anti–SARS-CoV-2 spike protein assay, it was not possible to differentiate past infection from immunization in our cohort. Therefore, the study ended early in January 2021. Due to rolling enrollment, participants had completed between 1 and 3 study visits by the end of the study period.

### Relationship of antibodies to subsequent COVID diagnosis

**Fig. 1.**
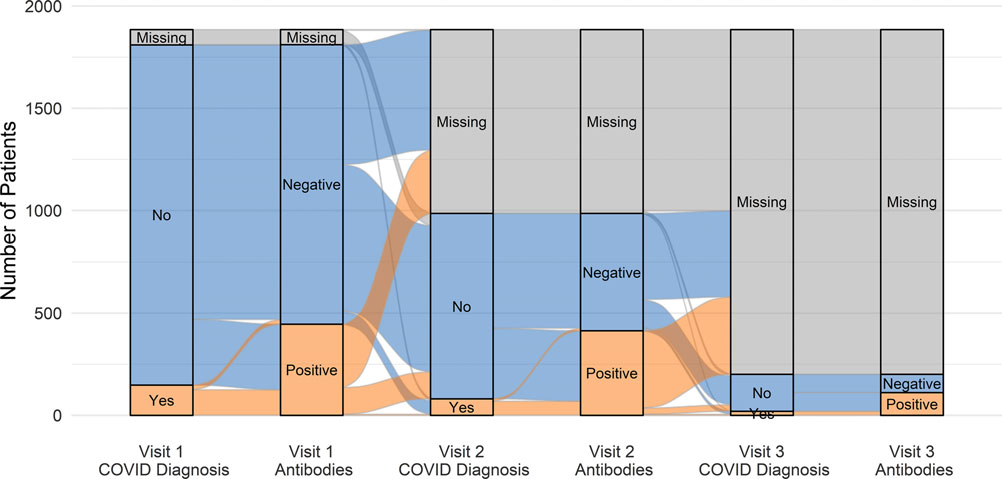
Evolution of SARS-CoV-2 antibody positivity and self-reported COVID-19 diagnosis over time. “Missing” refers to missed blood draws or questionnaires, and also to time points not yet reached by each participant.

In total, 1,885 participants enrolled in visit 1, and serologic data were available for 1,811. Overall seroprevalence in the cohort increased over time, from 25% at visit 1 to 55% at visit 3 (Table 1). Progression of participants through the study is shown in Figure 1 and Supplementary Figure 1. Overall, 446 participants had antibodies at visit 1 (baseline); 136 of them reported their diagnosis status between visits 1 and 2; and only 5 (4%) indicated they had been diagnosed with COVID-19. Of 1,365 participants with no antibodies at visit 1, 779 reported their diagnosis status between visits 1 and 2; 66 (8%) indicated they had been diagnosed with COVID-19 and 57 (86%) of these also developed antibodies. Of the 713 (92%) who reported no COVID-19 diagnosis, 208 (29%) nevertheless developed antibodies. Similarly, in the interval between visits 2 and 3, only 14 persistently seronegative participants reported a COVID-19 diagnosis, and all of them developed antibodies. Of the 132 reporting no diagnosis, 62 (47%) nevertheless developed antibodies.

**Table 1.** Prevalence of Self-Reported COVID-19 Exposure, Self-Reported COVID-19 Diagnosis, and SARS-CoV-2 Antibodies at Each Time Point

Variable	Visit 1 (baseline)	Visit 2 (60 days)	Visit 3 (120 days)
No. of participants with data	1,811	986	201
No. (%) with reported exposure	548 (30)	243 (25)	47 (23)
No. (%) with reported diagnosis	148 (8)	81 (8)	20 (10)
No. with diagnosis after an exposure	44	33	8
Median days from exposure to diagnosis	6	6	3.5
Antibodies present, no. (%)	446 (25)	414 (42)	111 (55)
Antibodies absent, no. (%)	1,365 (75)	572 (58)	90 (45)
No. (%) with antibodies after reported diagnosis	125(85)	69 (85)	20 (100)
No. (%) with no antibodies after reported diagnosis	23 (16)	11 (14)	0 (0)

### Persistence of antibodies

Of the 446 participants with antibodies at visit 1, 136 made it to visit 2 and 118 (87%) of them were still seropositive. Of those 136 participants at visit 2, only 10 made it to visit 3, and 100% were seropositive.

### Work location

The trend of increasing seroprevalence over time appeared not to differ among those reporting a main work location of COVID-19 unit, emergency department, obstetrics, long-term care facility, or other. The exception was the laboratory, with only 30% seroprevalence at visit 3 compared with a median of 74% for the other departments (Supplementary Fig. 2).

## Discussion

Our survey of patient care employees at a large healthcare system from June 2020 to January 2021 revealed increasing SARS-CoV-2 seroprevalence over a 6-month period. The number of employees with antibodies was higher than the number who reported an exposure or diagnosis at each time point. We speculate that this finding reflects increasing community transmission during this time, as well as the broad range of severity of SARS-CoV-2 infection including asymptomatic or subclinical cases. Previous studies have also found that seroprevalence among healthcare workers reflected seroprevalence in their surrounding communities.^3,4^ A study of healthcare workers in the Chicago area, also conducted from June 2020 to January 2021, likewise showed an increase in seropositivity over 6 months during a time of COVID-19 surge in the community.^5^ In our study, the percentage of participants reporting an interval COVID-19 diagnosis was similar at each time point, but the numbers were low overall. Larger studies have found that seropositive healthcare workers are less likely than seronegative healthcare workers to have a new COVID-19 diagnosis over a 6–7 month period.^6,7^

Our study had several limitations. Due to its remote design and streamlined data collection, we did not conduct SARS-CoV-2 testing or attempt to verify results. COVID-19 exposures, diagnoses, and work locations were all self-reported. The study ended early due to the availability of vaccines, leading to a small number of participants at visit 3. Nonetheless, this streamlined, pragmatic design showed that healthcare employees may be at higher risk for SARS-CoV-2 infection than they realize.

## Supplementary material

For supplementary material accompanying this paper visit https://doi.org/10.1017/ash.2022.9. click here to view supplementary material
