# Supplementary figures and images for: Severe acute respiratory syndrome coronavirus 2 (SARS-CoV-2) antibody prevalence in a healthcare worker population exceeded self-reported infection rates, 2020
Source: Antimicrob Steward Healthc Epidemiol. 2022 Feb 7;2(1):e21. doi: 10.1017/ash.2022.9 (PMC9614817; doi:10.1017/ash.2022.9)

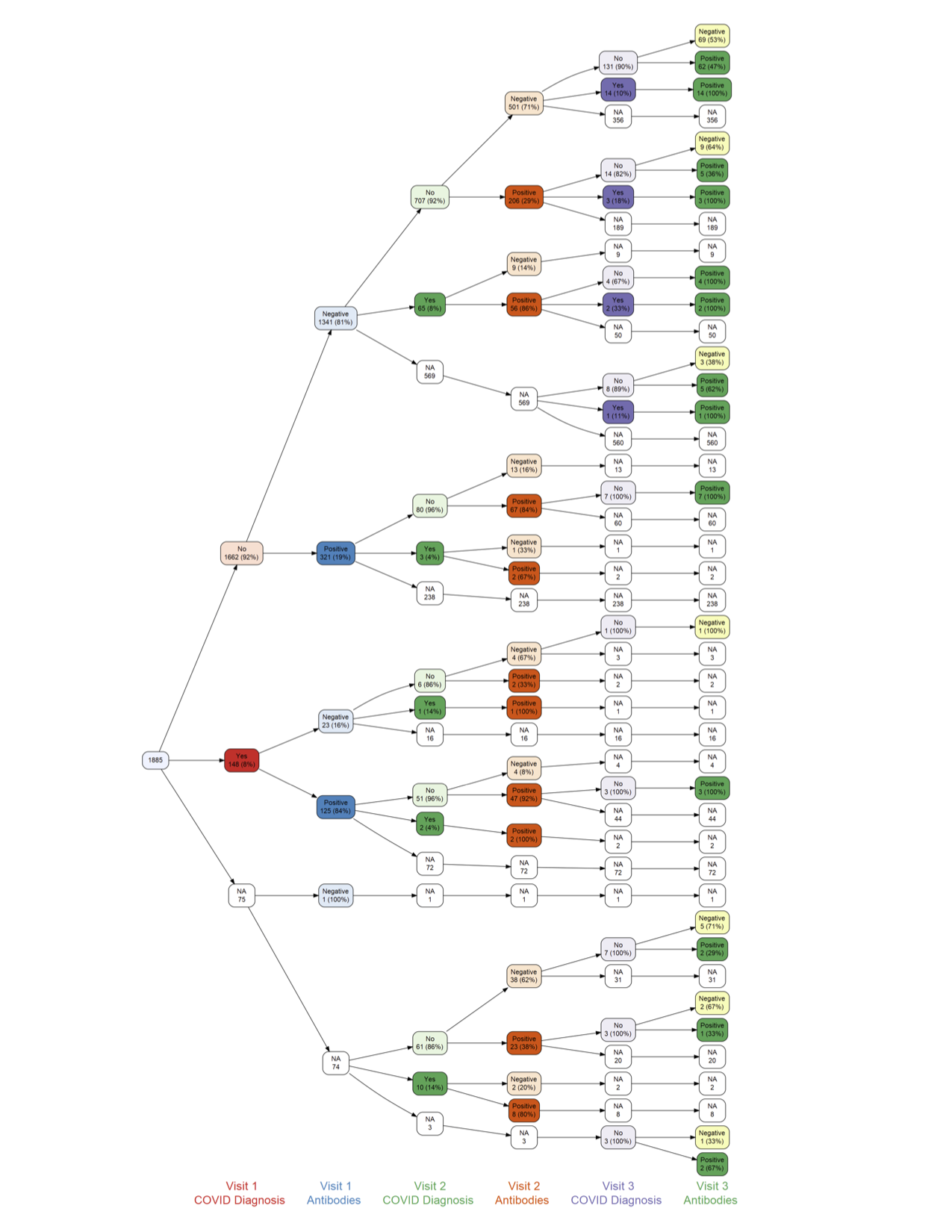

Supplement: Supplementary file 1 [file ashsup.zip › S2732494X22000092sup002.tiff]
